# Comparison of the Inhibitory Potential of Bavachalcone and Corylin against UDP-Glucuronosyltransferases

**DOI:** 10.1155/2014/958937

**Published:** 2014-04-16

**Authors:** Lina Shan, Shuman Yang, Gang Zhang, Dun Zhou, Zhenyu Qiu, Lei Tian, Hongxia Yuan, Yujun Feng, Xianbao Shi

**Affiliations:** ^1^The First Affiliated Hospital of Liaoning Medical University, Jinzhou 121001, China; ^2^Department of Environmental Health, University of Cincinnati, Cincinnati, OH 45267, USA; ^3^Department of Medicinal Chemistry, Virginia Commonwealth University, Richmond, VA 23219, USA; ^4^Maternal and Child Care Center of Qinhuangdao, Qinhuangdao 066000, China

## Abstract

Bavachalcone and corylin are two major bioactive compounds isolated from *Psoralea corylifolia* L., which has been widely used as traditional Chinese medicine for many years. As two antibiotic or anticancer drugs, bavachalcone and corylin are used in combination with other drugs; thus it is necessary to evaluate potential pharmacokinetic herb-drug interactions (HDI) of the two bioactive compounds. The aim of the present study was to compare the effects of liver UDP-glucuronosyltransferase (UGT) 1A1, UGT1A3, UGT1A7, UGT1A8, UGT 1A10, and UGT2B4 inhibited by bavachalcone and corylin. 4-Methylumbelliferone (4-MU) was used as a nonspecific “probe” substrate. Bavachalcone had stronger inhibition on UGT1A1 and UGT1A7 than corylin which did not inhibit UGT1A1, UGT1A3, UGT1A7, UGT1A8, UGT1A10, and UGT2B4. Data fitting using Dixon and Lineweaver-Burk plots demonstrated the noncompetitive inhibition of bavachalcone against UGT1A1 and UGT1A7-mediated 4-MU glucuronidation reaction. The values of inhibition kinetic parameters (Ki) were 5.41 **μ**M and 4.51 **μ**M for UGT1A1 and UGT1A7, respectively. The results of present study suggested that there was a possibility of UGT1A1 and UGT1A7 inhibition-based herb-drug interaction associated with bavachalcone and provided the basis for further in vivo studies to investigate the HDI potential between bavachalcone and UGT substrates.

## 1. Introduction

Fructus Psoraleae, derived from dried ripe fruit of* Psoralea corylifolia* L., has been commonly used as a traditional Chinese medicine for warming kidney, activating yang, promoting inspiration, and checking diarrhea [1]. It is listed in both the Chinese Pharmacopoeia and the “List of Herbal Materials that Can Be Used for Health Foods” under the Law on Food Hygiene by State Food and Drug Administration [2]. Modern pharmacological and clinical studies have shown that the extracts of Fructus Psoraleae possess different biological activities, such as antioxidative [3], antimicrobial [4], anti-inflammatory, and antitumor activities [5]. As antibiotic drug, Fructus Psoraleae attenuates lung inflammatory responses and repairs the impairment of pneumonia [6, 7]. In addition, Fructus Psoraleae was effective in the treatment of lung carcinoma [8, 9].

**Figure 1:**
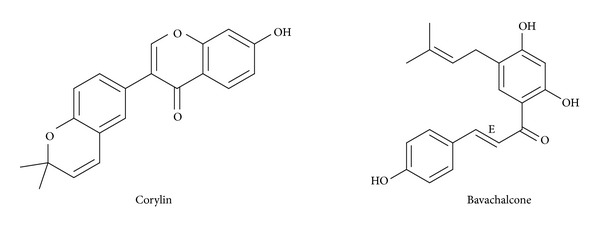
Structures of bavachalcone and corylin.

As two major compounds isolated from the seed of Fructus Psoraleae, bavachalcone and corylin (Figure 1) are extensively used in traditional medicine for the treatment of various diseases. For example, bavachalcone was found to inhibit osteoclastogenesis by interfering with the ERK and Akt signaling pathways and inhibit the production of Gram-positive bacteria [10, 11]. Similarly, corylin has strong antioxidant activities and can be used to treat diabetic complications [12, 13]. Although many biological and pharmacological activities of bavachalcone and corylin have been studied, further pharmacokinetic evaluation is conducted to understand their clinical efficiency and safety. Drug metabolizing enzyme- (DME-) catalyzed metabolic elimination significantly affects the concentration of drugs in plasma and therapeutic targets. Inhibition of the DMEs' activity can significantly increase the exposure of drugs, possibly resulting in the adverse effects of drugs, especially for the drugs with narrow therapeutic index. Accordingly, the study of potential interaction between herbal drugs and DME has received considerable attention over the last years [14]. However, more attention had been paid to cytochrome P450 (CYP450s) inhibition-based herb-drug interaction (HDI) because CYP450 is involved in most metabolisms of clinical drugs [15–17]. In contrast, other drug-metabolizing enzymes, such as phase II conjugating enzymes, have received less attention [18]. UGT-catalyzed glucuronidation reactions are responsible for the metabolism of approximately 35% of all drugs metabolized by phase II enzymes [19]. UGTs, important biochemical factors of cellular defense and detoxification, play an important role in the metabolism of many clinical drugs or their phase I metabolites [20]. Therefore, inhibition of UGT-catalyzed glucuronidation reactions is the key reason for testing clinical herb-drug interaction, because many xenobiotics have been demonstrated to have inhibitory effects against UGTs-catalyzed reactions, such as efavirenz [21], corydaline [22], and glycyrrhetinic acid [23].

At present, no study has evaluated the inhibitory effects of bavachalcone and corylin on UGT enzymes. In this study, the effects of bavachalcone and corylin on the activity of six major human UGTs were examined using recombinant human UGT supersomes to evaluate the possibility of bavachalcone or corylin-drug interactions.

## 2. Materials and Methods

### 2.1. Materials

Bavachalcone and corylin were purchased from Shifeng Corp. (Shanghai, China), and their purities were all above 98%. 4-Methylumbelliferone(4-MU), 4-methylumbelliferone-*β*-D-glucuronide(4-MUG), Tris-HCl, alamethicin, 7-hydroxycoumarin, and uridine 5′-diphosphoglucuronic acid (UDPGA) (trisodium salt) were purchased from Sigma-Aldrich (St. Louis, MO, USA). Recombinant human UGT supersomes (UGT1A1, UGT1A3, UGT1A7, UGT1A8, UGT1A10, and UGT2B4) expressed in baculovirus-infected insect cells were obtained from BD Gentest Corp. (Woburn, MA, USA). Solvents and other reagents were of HPLC grade.

### 2.2. Inhibition of 4-MU Glucuronidation Assay

The inhibition of bavachalcone and corylin to UGT isoforms activity was assessed using 4-MU as the nonspecific “probe” substrate. Incubation and analytical conditions have been described previously [24]. The mixture (200 *μ*L total volume) contained recombinant UGTs (final concentrations: 0.125, 0.05, 0.05, 0.025, 0.05, and 0.25 mg/mL for UGT1A1, UGT1A3, UGT1A7, UGT1A8, UGT1A10, and UGT2B4, resp.), 5 mM UDPGA, 5 mM MgCl_2_, 50 mM Tris-HCl buffer (pH = 7.4), and 4-MU in the absence or presence of different concentrations of bavachalcone and corylin. The concentrations of 4-MU were 100 *μ*M for UGT1A1, 1200 *μ*M for UGT1A3, 30 *μ*M for UGT1A7, 750 *μ*M for UGT1A8, 80 *μ*M for UGT1A10, and 1200 *μ*M for UGT2B4. Bavachalcone and corylin were dissolved in methanol and the final concentration of methanol was 0.5% (v/v). After a 5 min preincubation at 37°C, the UDPGA was added into the mixture to initiate the reaction. Incubation time was 120 min for UGT1A1, UGT1A3, UGT1A10, and UGT2B4 and 30 min for UGT1A7 and UGT1A8. The reactions were quenched by adding 100 *μ*L acetonitrile with 7-hydroxycoumarin (100 *μ*M) as internal standard. The mixture was centrifuged at 20,000 ×g for 20 min and 20 *μ*L of the supernatant was measured by UFLC. For each concentration, two samples in parallel were determined.

### 2.3. Ultrafast Liquid Chromatography (UFLC) Instrumentation and Conditions

The UFLC system (Shimadzu, Kyoto, Japan) contained a SCL-20A system controller, two LC-20AT pumps, a SIL-20A auto injector, and a SPD-20AV UV detector. Chromatographic separation was carried out using a Shim-pack XR-ODS column (2.0 × 75 mm, 2.2 *μ*M, Shimadzu) at flow rate of 0.5 mL/min and UV detector at 320 nm. The mobile phase consisted of acetonitrile (A) and H_2_O containing 0.5% (v/v) formic acid (B). The following gradient condition was used: 0–4.00 min, 95–50% B; 4.01–7.00 min, 5% B; and 7.01–10.00 min, 95% B.

### 2.4. Data Fitting for the Determination of Inhibition Type and Parameters (Ki)

If UGTs were strongly inhibited, half inhibition concentration (IC50) values were determined using various concentrations of bavachalcone (100, 80, 60, 40, 20, 10, 5, 1, and 0 *μ*M for UGT1A1 and 50, 20, 10, 6, 5, 3, 1, 0.5, and 0 *μ*M for UGT1A7) with previously described methods [24]. Dixon and Lineweaver-Burk plots were adapted to determine the inhibition type, and the second plot with the slopes from the Lineweaver-Burk plot versus the concentrations of components was utilized to calculate the Ki value.

## 3. Results

**Figure 2:**
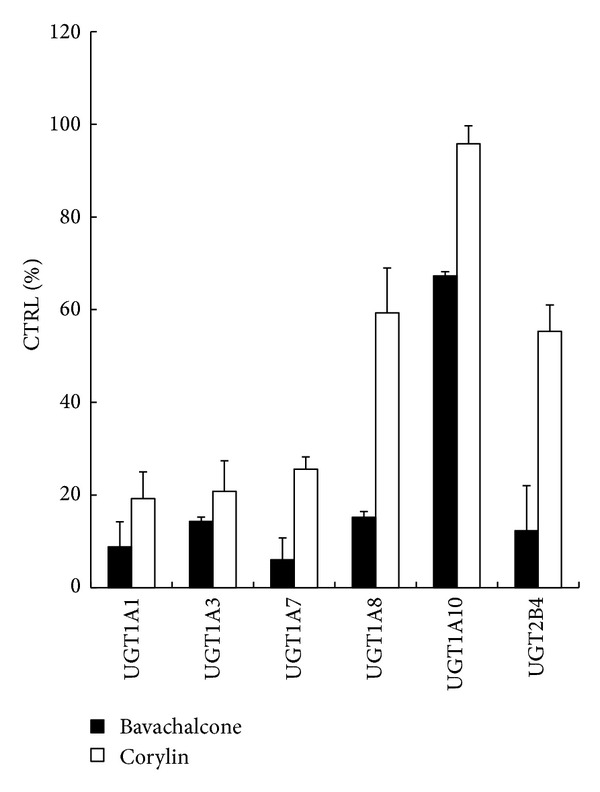
The inhibitory effects of bavachalcone and corylin on UGT1A1, UGT1A3, UGT1A7, UGT1A8, UGT1A10, and UGT2B4 isoforms. Recombinant UGT isoforms were used as enzyme sources. 4-MU was utilized as probe substrate. Incubation conditions were described in the method section.

**Figure 3:**
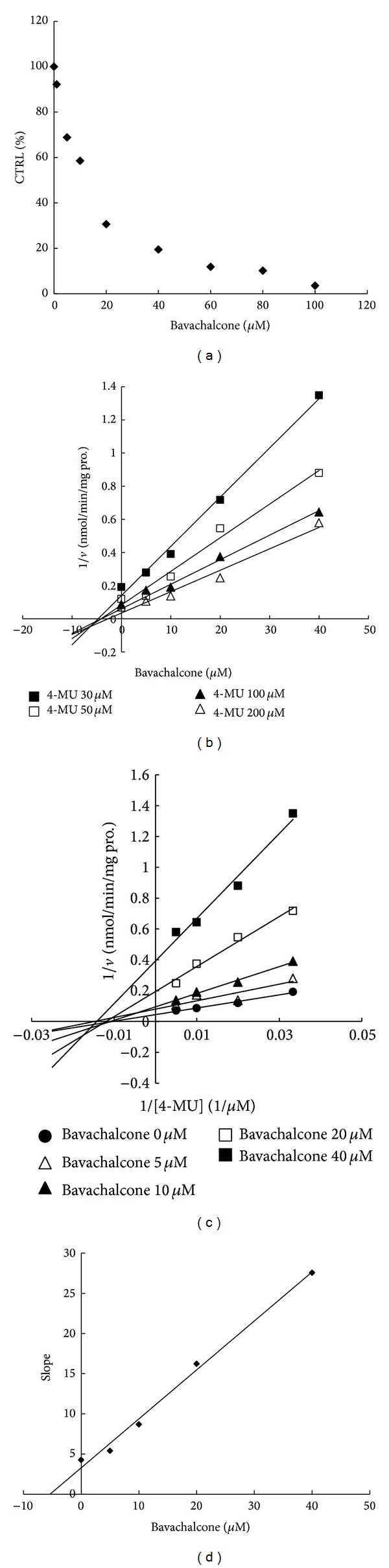
Determination of inhibition kinetic type and parameters (Ki) of UGT1A1 inhibited by bavachalcone. (a) Bavachalcone exhibits dose-dependent inhibition towards UGT1A1-catalyzed 4-MU glucuronidation. (b) Dixon plot of inhibitory effects of bavachalcone towards recombinant UGT1A1-catalyzed 4-MU glucuronidation. (c) Lineweaver-Burk plot of inhibitory effects of bavachalcone towards recombinant UGT1A1-catalyzed 4-MU glucuronidation. (d) Second plot of slopes from Lineweaver-Burk plot versus bavachalcone concentrations. Every data point represents the mean of two replicates.

**Figure 4:**
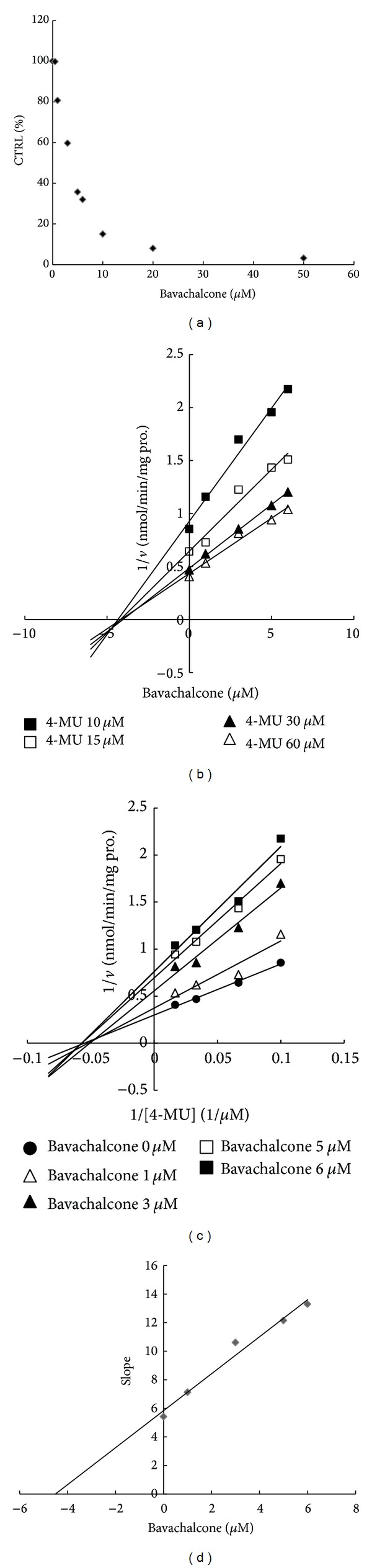
Determination of inhibition kinetic type and parameters (Ki) of UGT1A7 inhibited by bavachalcone. (a) Bavachalcone exhibits dose-dependent inhibition towards UGT1A7-catalyzed 4-MU glucuronidation. (b) Dixon plot of inhibitory effects of bavachalcone towards recombinant UGT1A7-catalyzed 4-MU glucuronidation. (c) Lineweaver-Burk plot of inhibitory effects of bavachalcone towards recombinant UGT1A7-catalyzed 4-MU glucuronidation. (d) Second plot of slopes from Lineweaver-Burk plot versus bavachalcone concentrations. Every data point represents the mean of two replicates.

The retention time of 4-MU, 4-MUG, and 7-hydroxycoumarin were 2.68, 3.19, and 3.66 min, respectively. As shown in Figure 2, 100 *μ*M of bavachalcone inhibited the activity of 4-MU glucuronidation by 91.2% (UGT1A1), 85.7% (UGT1A3), 94.0% (UGT1A7), 84.8% (1A8), 32.7% (UGT1A10), and 87.7% (UGT2B4), respectively. 100 *μ*M of corylin inhibited the activity of 4-MU glucuronidation by 80.8% (1A1), 79.2% (1A3), 74.4% (UGT1A7), 40.7% (1A8), 4.2% (UGT1A10), and 44.7% (UGT2B4), respectively. Furthermore, kinetic analysis was performed which activity has been inhibited by more than 90%. As shown in Figures 3(a) and 4(a), bavachalcone exhibited strongly concentration-dependent inhibitory behaviour against UGT1A1 and UGT1A7-catalyzed 4-MU glucuronidation, with IC50 values of 11.3 *μ*M for UGT1A1 and 3.6 *μ*M for UGT1A7, respectively. Dixon plot (Figures 3(b) and 4(b)) and Lineweaver-Burk plot (Figures 3(c) and 4(c)) showed that bavachalcone noncompetitively inhibited UGT1A1 and UGT1A7-mediated 4-MU glucuronidation, and the Ki value was calculated to be 5.41 *μ*M for UGT1A1 and 4.51 *μ*M for UGT1A7 (Figures 3(d) and 4(d)).

## 4. Discussion

It is necessary to determine how a potential drug inhibits or induces the enzymes involved in drug metabolism because these effects can lead to the molecular basis of interactions with other concomitantly administered drugs and may explain some toxic effects when a new drug is tested in vivo or in vitro [25]. Cytochromes P450 (CYP450s) and UDP-Glucuronosyltransferases (UGTs) are the major phase I and phase II drug metabolism enzymes. Therefore, many studies have focused on analyzing the effects of herbal components on major human CYP450s and UGTs in human hepatocytes. Metabolic behavior and inhibitory potential of Fructus Psoraleae have been attracting much attention of researchers. Psoralen and isopsoralen have been proven to have strong inhibitory potential against CYP1A2 [26]. It has been clearly demonstrated that CYP1A2, CYP2C9, CYP2C19, and CYP3A4 were the major CYP isoforms in liver microsomes (HLM) involved in the metabolism of bakuchiol and CYP2C19 has been shown to have the highest metabolic rate [27].

In this study, inhibitory effects of corylin and bavachalcone on six UGT isoforms were studied. Using a panel of recombinant human UGT isoforms, we found no potent inhibition of corylin against UGTs. In contrast, bavachalcone has been demonstrated to have a noncompetitive inhibition against UGT1A1 and UGT1A7 with the Ki values of 5.41 *μ*M and 4.51 *μ*M. UGT1A1 is responsible for the metabolism of many endogenous and exogenous substrates, including 15% drugs that have glucuronidation as a clearance mechanism of the top 200 drugs in the United States in 2002 [28]. For example, belinostat is an antitumor drug, and glucuronidation by UGT1A1 is the dominant pathway of the metabolic disposition [29]. The inhibition of UGT1A1 activity by bavachalcone might significantly affect the elimination of belinostat and then initiate the adverse effect when belinostat was coadministered with bavachalcone to treat tumor. UGT1A7 is an important phase II drug metabolism enzyme which is present only in the esophagus, stomach, and lung. UGT1A7 was involved in the metabolism of SN-38 and SN-38G [30]. So attention should be paid to bavachalcone-drug interactions when bavachalcone is used together with drugs metabolized by UGT1A1 or UGT1A7. The compounds containing a hydroxyl group on the benzene ring are very susceptible to metabolism catalyzed by phase II DMEs, including the UGTs [31]. Bavachalcone contains three hydroxyl groups and then corylin contains one hydroxyl group. This is possible reason that bavachalcone had stronger inhibition against UGT1A1 and UGT1A7 than corylin did. In addition, given the fact that many in vivo factors (absorption, distribution, metabolism, and excretion) could influence the in vitro-in vivo extrapolation (IVIVE), further in vivo experiments are required to evaluate the inhibition of drug-metabolizing enzymes by bavachalcone.

In conclusion, we have demonstrated the strong inhibitory effect of bavachalcone against UGT1A1 and UGT1A7. UGT1A1 and UGT1A7 are two important UGT isoforms involved in the metabolism of many drugs. Herb-drug interaction may exist when bavachalcone is coadministered with the clinical drugs which are able to be metabolized by UGT1A1 and UGT1A7.

## Supplementary Material

Supplementary Figure: Inhibitory effects of bavachalcone and corylin on important UGT1A1, UGT1A3, UGT1A7, UGT1A8, UGT1A10 and UGT2B4 isoforms. Recombinant UGT isoforms were used as enzyme sources. 4-MU was utilized as probe substrate. Incubation conditions were described in the experimental section.
