# Supplementary material for: Comparison of the Inhibitory Potential of Bavachalcone and Corylin against UDP-Glucuronosyltransferases
Source: Evid Based Complement Alternat Med. 2014 Apr 16;2014:958937. doi: 10.1155/2014/958937 (PMC4009204; doi:10.1155/2014/958937)
Supplement: Supplementary file 1 — Supplementary Figure: Inhibitory effects of bavachalcone and corylin on important UGT1A1, UGT1A3, UGT1A7, UGT1A8, UGT1A10 and UGT2B4 isoforms. Recombinant UGT isoforms were used as enzyme sources. 4-MU was utilized as probe substrate. Incubation conditions were described in the experimental section. [file 958937.f1.pdf]

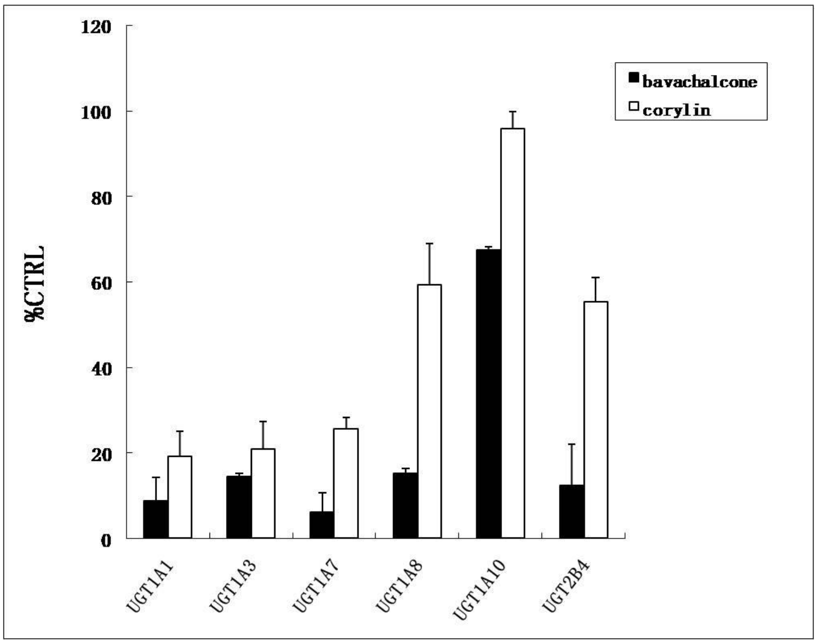

Fig. Inhibitory effects of bavachalcone and corylin on important UGT1A1, UGT1A3, UGT1A7, UGT1A8, UGT1A10 and UGT2B4 isoforms. Recombinant UGT isoforms were used as enzyme sources. 4-MU was utilized as probe substrate. Incubation conditions were described in the experimental section.
